# Retraction Note to: Pharmacological agents in development for diabetic macular edema

**DOI:** 10.1186/s40942-020-00271-8

**Published:** 2020-12-07

**Authors:** Mohammad Ali Sadiq, Muhammad Sohail Halim, Muha mmad Hassan, Neil Onghanseng, Irmak Karaca, Aniruddha Agarwal, Rubbia Afridi, Yasir J. Sepah, Diana V. Do, Quan Dong Nguyen

**Affiliations:** 1 Department of Ophthalmology, University of Louisville, Louisville, KY USA; 2 Byers Eye Institute, Stanford University, Palo Alto, CA 94303 USA; 3 Department of Ophthalmology, Ege University School of Medicine, Izmir, Turkey; 4 Advanced Eye Centre, Department of Ophthalmology, Postgraduate Institute of Medical Education and Research (PGIMER), Chandigarh, India; 5 Ocular Imaging Researchs and Reading Center (OIRRC), Sunnyvale, CA USA

## Retraction to: Int J Retin Vitr (2020) 6:29 10.1186/s40942-020-00234-z

The authors have retracted this article [1] for legal reasons. Therefore the contents of this article are no longer available. The authors have been invited to submit a new version of the article. All authors have agreed to this retraction.
